# Gout and the risk of myocardial infarction in older adults: a study of Medicare recipients

**DOI:** 10.1186/s13075-018-1606-z

**Published:** 2018-06-01

**Authors:** Jasvinder A. Singh, John D. Cleveland

**Affiliations:** 1 Medicine Service, VA Medical Center, 510, 20th Street South, FOT 805B, Birmingham, AL 35233 USA; 2 Department of Medicine at School of Medicine, University of Alabama at Birmingham, 1720 Second Ave. South, Birmingham, AL 35294-0022 USA; 3 Division of Epidemiology at School of Public Health, University of Alabama at Birmingham, 1720 Second Ave. South, Birmingham, AL 35294-0022 USA; 4 University of Alabama at Birmingham, Faculty Office Tower 805B, 510 20th Street S, Birmingham, AL 35294-0022 USA

**Keywords:** Gout, Myocardial infarction, Association, Risk, Elderly, Cardiac outcomes

## Abstract

**Background:**

Current evidence suggests that gout is independently associated with a higher risk of myocardial infarction (MI), but data in older adults at the highest risk of MI are lacking. Our objective was to examine whether gout is associated with a higher risk of incident MI in older adults.

**Methods:**

We assessed the 2006–2012 Medicare 5% claims data for the association of gout at baseline with the occurrence of a new (incident) MI during follow-up (no diagnosis of MI in the baseline period of at least 1 year), adjusting for patient demographics, medical comorbidity (Charlson–Romano index), and commonly used cardiovascular and gout medications, in a Cox proportional hazards model. Hazard ratios (HRs) and 95% confidence intervals (CIs) were calculated.

**Results:**

In a cohort of 1,733,613 eligible people, 14,279 developed incident MI: 13,029 MIs in people without gout and 1250 MIs in those with gout, with crude incident rates of 1.3 vs 4.1 per 1000 person-years, respectively. In multivariable-adjusted analyses, gout was significantly associated with a higher hazard of incident MI, with HR of 2.08 (95% CI 1.95, 2.21). Risk was minimally attenuated in sensitivity analyses that replaced the continuous Charlson–Romano index score with a categorical score or individual comorbidities, or expanding to a more sensitive diagnostic algorithm for incident MI, or additionally adjusting for obesity.

**Conclusions:**

Gout was independently associated with a higher risk of MI in the elderly, aged 65 years or older. The role of inflammatory and other pathways need to be explored as underlying mechanisms for this association.

## Background

Myocardial infarction (MI) is the most common, acute manifestation of coronary artery disease (CAD), which is the most common cardiovascular disease [1]. Although MI incidence decreased slightly from 1999 to 2008 [2], 790,000 Americans have MI each year [3]. MI is associated with high mortality rate [4] and significant health care costs [5]. Thus, MI is a significant public health problem with a huge burden on society and the health care system. Traditional risk factors for CAD are well known and include hypertension, hyperlipidemia, diabetes, smoking, family history, age, and postmenopausal status [6]. Lately, novel risk factors for CAD have been identified, such as obesity, lack of physical activity, and stress [6]. Recognition of novel risk factors for MI can improve our understanding of the disease as well as offer new therapeutic targets, in addition to the currently effective strategies for primary and secondary prevention and treatment of MI.

Gout was associated with 3-fold higher prevalence of the metabolic syndrome [7]. Gout was also associated with a higher prevalence of risk factors for MI, namely hyperlipidemia, hypertension, obesity, and diabetes [8]. Gout was associated with a 1.6-fold higher risk of incident CAD, after adjusting for systolic blood pressure, total cholesterol, alcohol intake, body mass index, and diabetes [9]; but in a case–control study, gout was not significantly associated with incident CAD after matching for age, sex, and medical practice, and the relative risk (RR) of incident CAD was 0.98 [10]. In a study of a British Columbia linked database of people with no history of ischemic disease, gout was associated with significantly increased relative risk of MI in women (RR 1.39 (95% CI 1.20, 1.61)), but not in men (RR 1.11; 95% CI 0.99, 1.23; *p* for interaction = 0.003); these analyses were adjusted for age, comorbidities, and prescription drug use [11]. In men from the MRFIT study, gout was associated with an adjusted odds ratio (OR) of 1.26 for MI [12]. In a study of an all-England national linked dataset of hospital admissions and death records from 1999 to 2011, compared to people without gout, gout was associated with a RR of 1.82 (95% CI 1.78, 1.85) for MI; findings were replicated in a similar dataset in the Oxford Record Linkage Study spanning 1963–1998 [13]. In Taiwanese population, gout was associated with a hazard ratio (HR) of 1.23 for MI, after adjustment for age, sex, and history of diabetes mellitus, hypertension, coronary heart disease, stroke, and end-stage renal disease [14]. Thus, gout seems to be associated with a higher risk of MI, although two studies reported no association overall or in men [10, 11].

Very few data exist with regards to the risk of MI associated with gout in people aged 65 years or older. This is a population at high risk of MI and poor outcome from MI. More than a third of all MIs occur in people aged > 70 years [15, 16] and more than 80% of deaths from MI occur in those 65 years or older [17]. The US population that is aged 65 years or older is predicted to grow from 34.4 million in 2000 to > 70 million in 2030 [18]. Given the public health burden of MI and associated mortality in the adults aged 65 years and older, potential cardiac risk associated with gout needs to be examined. Therefore, we aimed to assess whether gout was associated independently with a higher risk of incident MI in the elderly, and whether this association varied by CAD risk factors, including hypertension, hyperlipidemia, and diabetes.

## Methods

### Data sources and study sample

We used the claims data from the 5% Medicare sample from 2006 to 2012 [19]. Study eligibility criteria were being a Medicare beneficiary enrolled in Medicare fee-for-service (Parts A, B), and not enrolled in the Medicare Advantage Plan, and being resident in the USA during the study period, 2006–2012. The study was approved by the University of Alabama at Birmingham’s Institutional Review Board.

### Predictor of interest

A current diagnosis of gout was our main independent variable of interest. We required the presence of at least two claims at least 4 weeks apart with an International Classification of Diseases, ninth revision, common modification (ICD-9-CM) code of 274.xx, a validated approach with specificity and sensitivity of ≥ 90% [20]. The gout diagnosis had to present before the diagnosis of MI. Thus, all prevalent cases of gout at the beginning of the study window were included in the gout group and new gout cases during the study period were included, as long as the gout diagnosis preceded the diagnosis of MI.

### Independent variable/outcome of interest

The outcome of interest was incident MI, identified by the occurrence of two claims at least 4 weeks apart, each with an ICD-9-CM code for MI (410.x1), with no claims for MI in the baseline period of at least 1 year (excluding people with ICD-9-CM codes of 410 or 412 at baseline, 1/1/2015 to 12/31/2015), as described previously [21, 22]. This ICD-9 code-based approach has been shown to be valid, with positive predictive values > 90% [21, 23].

### Study covariates and confounders

We included several covariates and potential confounders in this study. Demographic variables included age (in years), gender, and race (White, Black, other), obtained from the Medicare denominator file and the beneficiary summary file. Medical comorbidity was assessed using the Charlson–Romano index, obtained from the inpatient and outpatient Medicare claim files. The Charlson–Romano index is a validated weighted comorbidity index, developed for claims data [24]; it was treated as a continuous score in the main model. We obtained data on the common cardiovascular drugs (statins, beta-blockers, diuretics, and angiotensin converting enzyme (ACE) inhibitors) and gout drugs (allopurinol, febuxostat) by including all prescription claims from the Medicare part D file. We included these drugs as surrogates for conditions they treat and/or disease severity and their protective or potentially protective effects related to the risk of MI.

### Statistical analyses

We compared characteristics of people with and without MI during the follow-up and calculated crude incidence rates per 1000 person-years. Patients contributed time to the control population prior to the diagnosis of gout. We assessed the association of gout with incident MI in multivariable-adjusted Cox regression analyses, that included all covariates already described, i.e., demographics, comorbidity, and the commonly used cardiovascular and gout medications. Hazard ratios (HRs) and 95% confidence intervals (CIs) were calculated.

Sensitivity analyses were performed by: replacing the continuous Charlson–Romano index score with a categorical score (model 2) or individual comorbidities (model 3; also included hypertension, hyperlipidemia, and coronary artery disease); additionally adjusting model 3 for obesity (ICD-9-CM code, 278.0); and expanding the diagnostic code for incident MI to 410 or 412 for incident MI, excluding people with code 410 or 412 at baseline (models 4–6), replicating models 1–3 for this more sensitive, less specific definition for incident MI. We also performed subgroup analyses by race, gender, and age as well as by MI risk factors.

## Results

Among 1,733,613 eligible people, 14,279 developed incident MI during the study follow-up. Of these, 13,030 cases occurred in people without gout (*n* = 1,639,534) and 1249 in those with gout (*n* = 94,809) (Table 1), with corresponding crude incident rates of 1.3 (13,030 cases/10,005,276 person-years) vs 4.1 (1249 cases/304,192 person-years) per 1000 person-years. Mean (standard deviation (SD)) time from gout diagnosis to the occurrence of incident MI was 2.3 years (1.7); median 1.9 years (interquartile range 0.8–3.5). When we compared to people without MI, those who had MI were older, more likely to be male, white, and have higher medical comorbidity, including a higher prevalence of cardiovascular disease, diabetes, connective tissue disease, and other comorbidities (Table 1).

**Table 1:** Demographic and clinical characteristics of people with and without myocardial infarction

	All episodes	Myocardial infarction during follow-up		*p* value
		No	Yes	
Total, *N*	1,733,613^a^	1,719,334	14,279	
Age, mean (SD)	75.3 (7.6)	75.3 (7.6)	77.0 (7.4)	< 0.0001
Gender, *N* (%)				< 0.0001
Male	734,540 (42.4%)	727,667 (42.3%)	6873 (48.1%)	
Female	999,073 (57.6%)	991,667 (57.7%)	7406 (51.9%)	
Race/ethnicity, *N* (%)				0.18
White	1,493,475 (86.1%)	1,481,232 (86.2%)	12,243 (85.7%)	
Black	142,284 (8.2%)	141,052 (8.2%)	1232 (8.6%)	
Other/unknown	97,854 (5.6%)	97,050 (5.6%)	804 (5.6%)	
Charlson–Romano comorbidity score				
0	913,332 (52.7%)	909,183 (52.9%)	4149 (29.1%)	< 0.0001
1	174,551 (10.1%)	172,711 (10.0%)	1840 (12.9%)	
≥ 2	645,730 (37.2%)	637,440 (37.1%)	8290 (58.1%)	
Charlson–Romano comorbidity score, mean (SD)	1.60 (2.39)	1.59 (2.38)	2.71 (2.79)	< 0.0001
Charlson–Romano comorbidities				
Myocardial infarction^b^	65,668 (3.8%)	64,977 (3.8%)	0 (0%)	< 0.0001
Heart failure	202,190 (11.7%)	199,011 (11.6%)	3179 (22.3%)	< 0.0001
Peripheral vascular disease	168,646 (9.7%)	165,601 (9.6%)	3045 (21.3%)	< 0.0001
Cerebrovascular disease	168,696 (9.7%)	166,159 (9.7%)	2537 (17.8%)	< 0.0001
Dementia	78,238 (4.5%)	77,698 (4.5%)	540 (3.8%)	< 0.0001
Chronic pulmonary disease	270,419 (15.6%)	267,014 (15.5%)	3405 (23.8%)	< 0.0001
Connective tissue disease	48,195 (2.8%)	47,571 (2.8%)	624 (4.4%)	< 0.0001
Peptic ulcer disease	32,778 (1.9%)	32,371 (1.9%)	407 (2.9%)	< 0.0001
Mild liver disease	8543 (0.49%)	8472 (0.49%)	71 (0.50%)	0.94
Diabetes	319,836 (18.4%)	314,635 (18.3%)	5201 (36.4%)	< 0.0001
Diabetes with end organ damage	94,249 (5.4%)	92,168 (5.4%)	2081 (14.6%)	< 0.0001
Hemiplegia	14,339 (0.83%)	14,164 (0.82%)	175 (1.2%)	< 0.0001
Renal failure/disease	59,280 (3.4%)	58,007 (3.4%)	1273 (8.9%)	< 0.0001
Any tumor, leukemia, or lymphoma	174,684 (10.1%)	172,971 (10.1%)	1713 (12.0%)	< 0.0001
Moderate or severe liver disease	2002 (0.12%)	1986 (0.12%)	16 (0.11%)	0.9
Metastatic cancer	18,009 (1.0%)	17,895 (1.0%)	114 (0.80%)	0.004
AIDS	549 (0.03%)	543 (0.03%)	6 (0.04%)	0.49
Hypertension	836,875 (48.3%)	827,183 (48.1%)	9692 (67.9%)	< 0.0001
Hyperlipidemia	602,546 (34.8%)	595,936 (34.7%)	6610 (46.3%)	< 0.0001
Coronary artery disease	302,088 (17.4%)	297,025 (17.3%)	5063 (35.5%)	< 0.0001
Obesity	36,034 (2.1%)	35,602 (2.1%)	432 (3.0%)	< 0.0001

^a^Met eligibility criteria and did not have MI in the baseline 365-day period; MI identified by the presence of two separate claims 4-weeks apart each with an ICD-9-CM code of 410.x1 during the study period of 2006–2012, with an exclusion of any person with ICD-9-CM code of 410.xx or 412.xx during the baseline 365-day period of 1/1/2005 to 12/31/2005

^b^Identified by the presence of ICD-9-CM code of 410.x or 412.x in the baseline 365-day period in 2005

*ICD-9-CM* International Classification of Diseases, ninth revision, common modification, *MI* myocardial infarction, *SD* standard deviation

In multivariable-adjusted analyses, gout was significantly associated with higher hazard of incident MI, with HR of 2.08 (95% CI 1.95, 2.21), which was minimally attenuated in sensitivity analyses that replaced the continuous Charlson–Romano score with a categorical score or individual comorbidities (models 2–3; Table 2). In addition, older age, male gender, white race, and higher comorbidity were each associated with a higher hazard of incident MI (Table 2).

**Table 2:** Association of gout and other risk factors with incident myocardial infarction

	Multivariable-adjusted^a^ model 1		Multivariable-adjusted^a^ model 2		Multivariable-adjusted^a^ model 3	
	HR (95% CI)	*p* value	HR (95% CI)	*p* value	HR (95% CI)	*p* value
Age (years)						
65 to < 75	Ref		Ref		Ref	
75 to < 85	**1.62 (1.56, 1.68)**	**< 0.0001**	**1.60 (1.54, 1.66)**	**< 0.0001**	**1.55 (1.49, 1.61)**	**< 0.0001**
≥ 85	**2.51 (2.39, 2.64)**	**< 0.0001**	**2.52 (2.39, 2.64)**	**< 0.0001**	**2.47 (2.35, 2.60)**	**< 0.0001**
Gender						
Male	Ref		Ref		Ref	
Female	**0.75 (0.73, 0.78)**	**< 0.0001**	**0.74 (0.72, 0.77)**	**< 0.0001**	**0.76 (0.74, 0.79)**	**< 0.0001**
Race						
White	Ref		Ref		Ref	
Black	1.00 (0.94, 1.06)	0.97	1.05 (0.99, 1.11)	0.10	0.95 (0.90, 1.01)	0.11
Other	**0.93 (0.86, 1.00)**	**0.038**	0.97 (0.90, 1.04)	0.35	**0.89 (0.83, 0.96)**	**0.002**
Charlson–Romano score, per unit change	**1.21 (1.21, 1.22)**	**< 0.0001**	N/A		N/A	
Charlson–Romano comorbidity score, *N* (%)						
0	N/A		Ref		N/A	
1			**2.20 (2.08, 2.33)**	**< 0.0001**		
≥ 2			**3.11 (2.99, 3.23)**	**< 0.0001**		
Gout	**2.08 (1.95, 2.21)**	**< 0.0001**	**2.14 (2.01, 2.27)**	**< 0.0001**	**1.79 (1.68, 1.90)**	**< 0.0001**

Bold data represent statistical significance, with *p* < 0.05

*CI* confidence interval, *HR* hazard ratio, *N/A* not applicable, *Ref* referent category

^a^Model 1 included Charlson–Romano score as a continuous variable; model 2 replaced it with categorized Charlson–Romano score; and model 3 replaced it with each of the 17 Charlson–Romano comorbidities. All models also adjusted for medications for cardiovascular diseases (statins, beta-blockers, diuretics, angiotensin converting enzyme inhibitors) and for urate-lowering therapies for gout (allopurinol, febuxostat)

Sensitivity analyses additionally adjusting model 3 for obesity led to minimal/no attenuation of HR from 1.79 (95% CI 1.69, 1.91) to 1.80 (95% CI 1.69, 1.91). Sensitivity analyses expanding the ICD-9-CM code for incident MI to a more sensitive and less specific diagnostic code algorithm (code 410 or 412) revealed slightly lower HRs of incident CAD related to gout in models corresponding to the three models as shown in Table 2 above: 1.85 (95% CI 1.79, 1.92; model 4), 1.83 (95% CI 1.77, 1.89; model 5), and 1.59 (95% CI 1.54, 1.65; model 6).

In subgroup analyses, hazard ratios of gout with incident MI were higher in the absence of hypertension, hyperlipidemia, diabetes, or heart failure (ranging from 2.2 to 3.0) vs hazard ratios in those with each of these comorbidities (ranging from 1.6 to 1.7) (Table 3), differences that were both statistically significant and seemed clinically meaningful. Similarly, the hazard ratios for MI with gout were 2.2 and 1.7 in those without vs with CAD (Table 3). We noted minor differences by age, gender, and race which were only statistically significant, except for difference in HR between white and black race, which also seemed potentially clinically meaningful (2.02 vs 2.49) (Table 3).

**Table 3:** Association of gout with MI, in predefined subgroup analyses

	Multivariable-adjusted model 1		Multivariable-adjusted model 1		Multivariable-adjusted model 1	
	HR (95% CI)	*p* value	HR (95% CI)	*p* value	HR (95% CI)	*p* value
	Black		White		Other race	
Gout	**2.49 (2.10, 2.96)**	**< 0.0001**	**2.02 (1.89, 2.16)**	**< 0.0001**	**2.05 (1.59, 2.65)**	**< 0.0001**
	Female		Male			
Gout	**2.13 (1.94, 2.35)**	**< 0.0001**	**2.04 (1.89, 2.21)**	**< 0.0001**		
	Age 65–75 years		Age 75–85 years		Age > 85 years	
Gout	**2.09 (1.90, 2.31)**	**< 0.0001**	**1.89 (1.73, 2.08)**	**< 0.0001**	**2.50 (2.15, 2.91)**	**< 0.0001**
	No hypertension		Hypertension			
Gout	**3.00 (2.65, 3.40)**	**< 0.0001**	**1.73 (1.61, 1.85)**	**< 0.0001**		
	No diabetes		Diabetes			
Gout	**2.35 (2.17, 2.55)**	**< 0.0001**	**1.64 (1.49, 1.80)**	**< 0.0001**		
	No hyperlipidemia		Hyperlipidemia			
Gout	**2.45 (2.24, 2.69)**	**< 0.0001**	**1.74 (1.60, 1.90)**	**< 0.0001**		
	No heart failure		Heart failure			
Gout	**2.20 (2.04, 2.36)**	**< 0.0001**	**1.73 (1.55, 1.93)**	**< 0.0001**		
	No CAD		CAD			
Gout	**2.18 (2.01, 2.37)**	**< 0.0001**	**1.74 (1.59, 1.91)**	**< 0.0001**		

Race × gout, *p* = 0.10

Age × gout, *p* < 0.05

Gender × gout, *p* = 0.017

Hypertension × gout, *p* < 0.0001

Diabetes × gout, *p* < 0.0001

Heart failure × gout, *p* < 0.0001

Hyperlipidemia × gout, *p* < 0.0001

CAD × gout, *p* < 0.0001

Bold data represent significant HRs with *p* < 0.05

*HR* hazard ratio, *CI* confidence interval, *CAD* coronary artery disease

## Discussion

We noted a strong, robust, independent association of gout with incident MI in adults aged 65 years or older, confirmed in multiple sensitivity analyses for the main analysis (models 2–3); confirmed further when we expanded the ICD-9 code from 410.x1 (models 1-3) to a more sensitive definition using ICD-9 codes 410 or 412 (models 4–6). This is an important finding that merits further discussion. In previous studies in the general population, gout was associated with a higher risk/hazard ratio of incident MI ranging from 1.23-fold to 1.82-fold in some studies [11–14], but not associated with higher risk in others [10, 25]. Studies differed in setting (cohort vs record-linkage), population (men vs women vs both), and confounders adjusted (cardiovascular risk factors included vs not). Our hazard ratio estimates in models 1–3 ranging from 1.79 to 2.08 for adults aged 65 years or older are slightly higher than previous estimates, since we were examining an older patient population.

Our study examined men and women 65 aged years or older in the USA, controlling for cardiovascular risk factors among other important factors. Estimates were robust in sensitivity analyses. The implications of our study are several. Using a representative US patient population, we provided estimates for gout and incident MI, which adds clarity to this important clinical question. An increased risk of incident MI in people with gout raises a question regarding the role of chronic inflammation and IL-1β pathways (via NLRP3 inflammasome activation) and CRP, hallmarks of gout [26, 27], in the pathogenesis of MI. The IL-1β pathway is shown to be important in the pathogenesis of MI [28–35] and as a downstream effect increases IL-6 levels, a potential causal pathway for atherothrombosis [36, 37]. Various pathogenic mechanisms common in people with CAD risk factors, such as cholesterol crystals, tissue hypoxia, and abnormal arterial flow patterns, can promote the activation of the NLRP3 inflammasome [38–41], which then activates IL-1β. CRP is elevated in CAD and contributes directly to atherosclerosis via leukocyte activation and endothelial dysfunction [42–44]. Our study findings generate the hypothesis that inflammatory pathways may be activated in the atherosclerotic plaque, which may then lead to MI. People with gout have upregulation of these inflammatory pathways, which might explain the increased risk of MI in gout, at least partially.  The role of key mechanisms of increased atherosclerosis in gout needs to be examined in basic and translational studies.

We noted that the association of gout with incident MI was stronger in people without each CAD risk factor (hypertension, hyperlipidemia, or diabetes) (HR 2.3–3.0) than in the presence of each CAD risk factor respectively (HR 1.6–1.7). This indicates that in patients with known CAD/MI risk factors, gout contributes much less to the risk of incident MI. Most differences were not only statistically significant, but also clinically meaningful. We also noted a similar trend in presence vs absence of heart failure and CAD. This observation is similar to that noted by Kuo et al. previously in a Taiwanese study [14]. We speculate two potential reasons for this observation: gout is associated with a 3-fold higher prevalence of the metabolic syndrome [7] that has features of hyperlipidemia, hyperglycemia, or obesity, and gout may be an early clinical manifestation of the metabolic syndrome; and episodic inflammation characteristic of gout flares may increase the MI risk, especially in those without other CAD risk factors.

In a subgroup analysis, we found that gender made little difference to the association of gout with incident MI in the elderly. Previous studies found higher risk of MI with gout in UK women compared to men (2.08 (95% CI 2.01, 2.16) vs 1.73 (95% CI 1.69, 1.77), respectively) [13], considering all age groups (mean age 70 years), or a trend of higher risk of MI with gout in Canadian women compared to men (1.11 (95% CI 0.99, 1.23); *p* = 0.003 for interaction by gender) [11] in the elderly with a mean age of 75 years. The mean age of people in our study is similar to these studies at 73 years. The reasons for differences in the findings is likely related to differences in country setting (USA vs UK vs Canada), confounders adjusted in the analyses (cardiovascular diseases, cardiovascular medications, and gout medications vs neither vs cardiovascular medications only), the outcome definition, the underlying conditions (none vs. none vs. musculoskeletal disease), study sample (all Medicare recipients vs. all hospital admissions for gout vs. all elderly) and the population examined in each study (limited to only people aged 65 years or older vs all ages vs. women 65 years or older).

Our study has several limitations, which must be considered while interpreting the findings. We used data from Americans aged 65 years or older, and therefore the generalizability of these findings to younger people is uncertain. Diagnostic misclassification may have occurred despite our use of validated algorithms; this would bias our study findings toward the null, making our findings conservative (i.e., we may have missed some associations). Residual confounding is still possible, given an observational study design, despite the fact that we controlled for several potential confounders. We adjusted for several potential confounders including cardiovascular drugs, but we did not adjust for aspirin, nonsteroidal anti-inflammatory drug (NSAID) use, alcohol use, smoking, or exercise, which may have led to some residual confounding. Adjustment for aspirin and NSAID use was considered but not done, since most NSAID and aspirin use in this age group is over the counter rather than prescription use [45, 46]. Over-the-counter medication use is not captured in the Medicare claims data, which would introduce misclassification bias. We are also unaware of the differential rate/pattern of the use of aspirin or NSAID by gout status, for primary or secondary prevention of CAD. Our study has several strengths. Inclusion of medications for cardiovascular disease and gout strengthens the analyses, since these medications might be imperfect surrogates of disease severity which Medicare data lack and have independent protective effects related to MI risk, but also in some cases may be indicative of the presence of a disease in the absence of an ICD-9-CM code and may reduce misclassification bias. We used a representative sample of US adults aged 65 years or older, had an adequate number of events for analyses, and conducted multiple sensitivity analyses to test the robustness of findings.

## Conclusions

This study showed an association of gout with incident MI in adults aged 65 years or older, independent of the traditional CAD risk factors. The MI risk associated with gout was stronger in people without CAD risk factors compared to people with CAD risk factors, and the risk was increased 2-fold or higher in both groups. Chronic inflammation, a hallmark of gout, is implicated in the pathogenesis of incident MI [28]. Future studies should evaluate the mechanisms for this disease association, and evaluate to what extent this association is due to chronic inflammation versus other potential pathways.

## Abbreviations

ACE: Angiotensin converting enzyme
CAD: Coronary artery disease
CRP: C-reactive protein
ICD-9-CM: International Classification of Diseases, ninth revision, common modification
MI: Myocardial infarction
SD: Standard deviation
